# Lack of replication of higher genetic risk load in men than in women with systemic lupus erythematosus

**DOI:** 10.1186/ar4585

**Published:** 2014-06-19

**Authors:** Elisa Alonso-Perez, Marian Suarez-Gestal, Manuel Calaza, Francisco J Blanco, Ana Suarez, Maria Jose Santos, Chryssa Papasteriades, Patricia Carreira, Rudolf Pullmann, Josep Ordi-Ros, Maurizio Marchini, Fotini N Skopouli, Marc Bijl, Nadia Barrizone, Gian Domenico Sebastiani, Sergio Migliaresi, Torsten Witte, Bernard R Lauwerys, Attila Kovacs, Sarka Ruzickova, Juan J Gomez-Reino, Antonio Gonzalez

**Affiliations:** 1 Research laboratory 10 and Rheumatology Unit, Health Research Institute–Clinic Hospital of Santiago, Choupana s/n, Santiago de Compostela 15706, Spain; 2 Rheumatology Service, INIBIC–University Hospital Complex A Coruña, Jubias de Arriba 84, A Coruña 15006, Spain; 3 Department of Functional Biology, University of Oviedo, Julián Clavería s/n, Oviedo 33006, Spain; 4 Rheumatology Department, Hospital Garcia de Orta and Rheumatology Research Unit, Molecular Medicine Institute, Prof Egas Moniz s/n, Lisbon 1649-028, Portugal; 5 Department of Histocompatibility and Immunology, Evangelismos Hospital, Ipsilantou Str 45-47, Athens 10675, Greece; 6 Rheumatology Department, 12th of October Hospital, Av Andalucía s/n, Madrid 28041, Spain; 7 Institute of Clinical Biochemistry, Martin Faculty Hospital and Jessenius Medical Faculty, Kollárova 2, Martin 036 59, Slovakia; 8 Internal Medicine and Research Laboratory in Autoimmune Diseases, Hospital Vall d’Hebron, Passeig Vall d’Hebron 119-129, Barcelona 08035, Spain; 9 Referral Center for Systemic Autoimmune Diseases, Foundation IRCCS General Hospital and University of Milan, Via Francesco Sforza 35, Milan 20122, Italy; 10 Euroclinic of Athens, Athanasiadou str 7-9, 11521 Athens, Greece; 11 Department of Internal Medicine and Rheumatology, Martini Hospital, Van Swietenplein 1, Groningen 9728, the Netherlands; 12 Department of Medical Sciences and Interdisciplinary Research Center of Autoimmune Diseases (IRCAD), University of Eastern Piedmont Amedeo Avogadro, 28100 Novara, Italy; 13 UOC Rheumatology, Reumatologia, San Camillo-Forlanini Hospital, Rome, Italy; 14 Rheumatology Unit, Second University of Naples, 81100 Naples, Italy; 15 Division of Clinical Immunology, Department of Internal Medicine, Hannover Medical School, 30625 Hannover, Germany; 16 Saint-Luc University Clinic and Catholic University of Leuven, Brussels, Belgium; 17 Department of Rheumatology, Hospital of Hungarian State Railways, 5000 Verseghy u6-8, Szolnok, Hungary; 18 Institute of Biotechnology, Academy of Sciences of the Czech Republic, Vídeňská 1083 142 20, Prague, Czech Republic; 19 Department of Medicine, University of Santiago de Compostela, Calle Choupana s/n, Santiago de Compostela 15706, Spain

## Abstract

**Introduction:**

We aimed to replicate a recent study which showed higher genetic risk load at 15 loci in men than in women with systemic lupus erythematosus (SLE). This difference was very significant, and it was interpreted as indicating that men require more genetic susceptibility than women to develop SLE.

**Methods:**

Nineteen SLE-associated loci (thirteen of which are shared with the previous study) were analyzed in 1,457 SLE patients and 1,728 healthy controls of European ancestry. Genetic risk load was calculated as sex-specific sum genetic risk scores (GRS_s_).

**Results:**

Our results did not replicate those of the previous study at either the level of individual loci or the global level of GRS_s_. GRS_s_ were larger in women than in men (4.20 ± 1.07 in women vs. 3.27 ± 0.98 in men). This very significant difference (*P* < 10^−16^) was more dependent on the six new loci not included in the previous study (59% of the difference) than on the thirteen loci that are shared (the remaining 41%). However, the 13 shared loci also showed a higher genetic risk load in women than in men in our study (*P* = 6.6 × 10^−7^), suggesting that heterogeneity of participants, in addition to different loci, contributed to the opposite results.

**Conclusion:**

Our results show the lack of a clear trend toward higher genetic risk in one of the sexes for the analyzed SLE loci. They also highlight several limitations of assessments of genetic risk load, including the possibility of ascertainment bias with loci discovered in studies that have included mainly women.

## Introduction

The large sex bias in systemic lupus erythematosus (SLE) incidence has not been satisfactorily explained, although sex hormones, cell microchimerism and chromosome X dosage seem to be involved [1-4]. There are other aspects of SLE in addition to incidence in which patient sex is important [3-6]. They include an increased prevalence of renal disease, serositis and discoid lupus and a decrease in the mucocutaneous manifestations in men. Other differences between women and men have been reported with less consistency between studies as a higher prevalence of thrombocytopenia, anti–double-stranded DNA antibodies, decreased C3 and larger organ damage accrual in men than in women with SLE.

The difference in incidence is maximal during the fertile years. It is preceded by a more similar incidence in girls and boys before puberty and followed by a return toward equilibrium in incidence between the sexes in the eldery. This pattern is a powerful suggestion of the effect of sex hormones in SLE pathogenesis [1-4]. This hypothesis is supported by the results of estrogen suppression and estrogen administration experiments in SLE animal models, but the evidence is less clear in humans. There are not abnormal levels of sex hormones in most SLE patients of either sex. Only hyperprolactinemia is commonly found, but with uncertain involvement in disease pathogenesis. In addition, sex hormone therapy is associated with either no or very small increases in SLE or its severity. Other factors contributing to the increased SLE incidence in women could be lymphoid cell microchimerism as a consequence of pregnancy [1]. The incompatibility between the two lymphoid populations gives rise to autoimmunity in animal models, but the causal relationship in SLE patients is less clear, as microchimerism in damaged organs seems to increase as a consequence of tissue repair [7]. A third factor explaining the sexual dimorphism of SLE is suspected to reside in differences between the sex chromosomes [1-4]. The hypothesized mechanism involves a dose excess of genes promoting autoimmunity in women due to noninactivated X chromosomes. This hypothesis has received strong support based on the demonstration of an increased incidence of SLE in patients with Klinefelter’s syndrome who have an external male phenotype but carry the XXY sex chromosomes [8].

More recently, evidence supporting the possibility of a second genetic factor has been reported. Researchers who analyzed a very large collection of patients of European ancestry showed that the genetic risk load was very significantly larger in men than in women (*P* = 4.52 × 10^−8^), based on the sum genetic risk score (GRS_s_) of 15 SLE loci [9]. This result was interpreted as meaning that men require more genetic susceptibility than women to develop SLE. The difference in genetic risk load in that study was very dependent on two human leukocyte antigen (HLA) loci of the fifteen loci included. This was shown by the lack of significant difference between men and women when the two HLA single-nucleotide polymorphisms (SNPs) were excluded (*P* = 0.3). Our interest in the implications of a higher genetic risk load in men led us to replicate this previous study, but our results are contrary to those reported. We found that genetic risk load was very significantly higher in women than in men with SLE.

## Material and methods

### Clinical and genotype data

The participant samples used in this study have already been described [10-12]. Briefly, 17 recruiting centers in 10 European countries were asked for about 100 SLE patients who met the American College of Rheumatology criteria [13] and a similar number of healthy controls, all of whom have uniform ancestry from the country of recruitment. In our present study, samples overlapping with the Hughes *et al*. [9] report were considered separately. Recruiters asked each participant for his or her ancestry, and only those reporting uniform known ancestry from the respective countries were included. In addition, we used six top ancestry-informative markers for European population differentiation to determine whether there were differences between cases and controls from each recruitment center. Samples from two centers were excluded because cases and controls showed significant differences at any of the six top ancestry-informative markers analyzed, as described previously [11]. This selection process left us with a total of 1,457 SLE patients and 1,728 healthy controls from 15 collections and 8 countries. A large fraction of these samples did not overlap with the previous study of SLE genetic risk load. Specifically, the nonoverlapping samples were from 1,124 SLE patients and 1,422 healthy controls from 11 collections and 6 countries. All participants provided their written informed consent as approved by the respective local ethics committees: the Ethics Committee for Clinical Research of Galicia for samples in Santiago and Corunna, the Regional Ethics Committee for Clinical Research of Asturias for samples in Oviedo, the Garcia de Orta Hospital Ethics Committee for samples in Lisbon, the Ethics Committee of Evangelismos Athens General Hospital and Scientific Committee of the Euroclinic Hospital for samples in Athens, the Ethics Committee for Clinical Research of the Doce de Octubre University Hospital for samples in Madrid, the Ethical Committee of the Jessenius Medical Faculty for samples in Martin, the Ethics Committee for Clinical Research of the Vall d’Hebron Hospital for samples in Barcelona, the Ethics Committee of the Fondazione IRCCS Cà Granda Ospedale Maggiore Policlinico for samples in Milan, the Ethical Committee of the University Medical Center Groningen for samples in Groningen, the Ethics Committee Azienda Ospedaliera San Camillo–Forlanini for samples in Rome, the Ethical Committee of the Second University of Naples for samples in Naples, the Ethics Commission of the Medical School of Hannover for samples in Hannover, the Commission d’Ethique Biomédicale Hospitalo–Facultaire de l’Université Catholique de Louvain for samples in Brussels, the Institutional Ethics Committee of the Hospital of Hungarian State Railways for Hungarian samples and the Ethical Committee of the Institute of Rheumatology for samples in Prague. The overall study was approved by the Comite Etico de Investigacion Clinica de Galicia. The clinical characteristics of the patients were obtained at the same time. These data included the American College of Rheumatology classification criteria met, sex, age at time of disease onset and time to follow-up. The genotypes of 26 SNPs showing the greatest association at SLE loci identified in large studies [14-23] were available to us from previous studies [11,12,24]. All have been genotyped in the same laboratory and with the same technology: single-base extension using the SNaPshot Multiplex Kit (Applied Biosystems, Foster City, CA, USA), and have passed quality control filters. Nineteen of these SNPs were selected for analysis because they have reached association with SLE at the genome-wide association study significant level (*P* < 5 x 10^-8^) and because they were independently associated with SLE in our samples. Thirteen of these nineteen loci are shared with the fifteen SNPs included in the GRS_s_ reported by Hughes *et al*. [9], and the other six loci have not been analyzed previously in the context of the sex differential genetic risk load. The thirteen loci shared between their studies and ours are listed in the top rows of Table 1, and the six new loci are listed in the bottom rows. Eleven of the thirteen shared loci were studied with the same SNPs used by Hughes *et al.*[9]; the remaining two loci, rs17266594 in *BANK1* and rs10488631 in *IRF5,* were studied with highly correlated SNPs (*r*^2^ > 0.9 for rs10516487 and rs2070197, respectively, according to the HapMap data for the European population).

**Table 1:** **List of the 19 systemic lupus erythematosus loci included in this study and their association with systemic lupus erythematosus in our samples**^**a**^

**SNP**	**Gene locus**^**b**^	**Chromosome**	**Position**	**SLE**^**c**^	**Control**^**c**^	**OR (95% CI)**	***P***-**value**
rs2476601	*PTPN22*	1	114377568	0.099	0.074	1.37 (1.15 to 1.63)	4.3 × 10^−4^
rs1801274	*FCGR2A*	1	161479745	0.509	0.475	1.15 (1.04 to 1.27)	6.2 × 10^−3^
rs2205960	*TNFSF4*	1	173191475	0.264	0.217	1.29 (1.15 to 1.45)	1.0 × 10^−5^
rs7574865	*STAT4*	2	191964633	0.323	0.233	1.57 (1.41 to 1.76)	1.2 × 10^−15^
rs6445975	*PXK*	3	58370177	0.268	0.231	1.22 (1.09 to 1.37)	6.2 × 10^−4^
rs17266594	*BANK1*^d^	4	98488450	0.749	0.711	1.21 (1.09 to 1.36)	6.6 × 10^−4^
rs3131379	*MSH5*	6	31721033	0.145	0.072	2.18 (1.85 to 2.58)	4.2 × 10^−21^
rs729302	*IRF5*	7	122930164	0.744	0.690	1.31 (1.17 to 1.46)	2.5 × 10^−6^
rs10488631	*IRF5*^d^	7	128594183	0.183	0.101	1.99 (1.72 to 2.31)	1.8 × 10^−20^
rs10954213	*IRF5*	7	128589427	0.680	0.641	1.19 (1.07 to 1.32)	1.1 × 10^−3^
rs13277113	*C8orf13-BLK*	8	11349186	0.308	0.247	1.35 (1.21 to 1.51)	8.1 × 10^−8^
rs4963128	*KIAA1542*	11	589564	0.698	0.656	1.21 (1.09 to 1.34)	5.0 × 10^−4^
rs1143679	*ITGAM*	16	31276811	0.236	0.158	1.65 (1.45 to 1.87)	7.0 × 10^−15^
rs2304256	*TYK2*	19	10475652	0.766	0.725	1.24 (1.11 to 1.39)	2.0 × 10^−4^
rs5754217	*UBE2L3*	22	21939675	0.253	0.216	1.24 (1.10 to 1.39)	3.6 × 10^−4^
rs2230926	*TNFAIP3*	6	138196066	0.074	0.040	1.89 (1.52 to 2.35)	8.5 × 10^−9^
rs573775	*ATG5*	6	106764866	0.297	0.267	1.16 (1.04 to 1.29)	8.8 × 10^−3^
rs2187668	*HLA-DQA1*	6	32605884	0.200	0.106	2.11 (1.83 to 2.43)	1.2 × 10^−25^
rs10798269	*1q25.1*	1	173309713	0.729	0.685	1.24 (1.11 to 1.38)	1.3 × 10^−4^

^a^SLE, Systemic lupus erythematosus; SNP, Single-nucleotide polymorphism. ^b^The 13 first loci are shared with those studied by Hughes *et al*. [1]. ^c^Risk allele frequencies in patients with SLE (*n* = 1,457) and healthy controls (*n* = 1,728). ^d^*r*^2^ = 0.94 with rs10516487 used for *BANK1* by Hughes *et al*. [9] and *r*^2^ = 0.94 for rs2070197 used for *IRF5* by Hughes *et al*. [9].

### Analysis of sex-specific genetic risk load

We compared SNP allele frequencies between SLE patients and controls, women together with men and women and men separately. Also, allele frequencies were compared between women and men with SLE. These comparisons were done by performing χ^2^ tests. GRS_s_ were obtained by applying the same approach used by Hughes *et al.*[9]. In brief, the number of risk alleles (none, one or two) carried by a patient at each locus was multiplied by the natural logarithm of the sex-specific odds ratio (OR) of that locus. The products corresponding to all loci were summed to obtain the GRS_s_ for this patient. Only patients with valid genotypes at all loci were included in this analysis (1,247 women and 125 men with SLE, of whom 981 women and 96 men were nonoverlapping with those in the previous study). GRS_s_ corresponding to women and men were compared using Student’s *t*-test. These analyses were done in a customized version of Statistica 7.0 software (StatSoft, Tulsa, OK, USA). Power analysis was done using the Power and Sample Size software [25].

## Results

The 19 SNPs that we investigated were significantly different between SLE patients and controls in the unstratified analysis of our 1,457 SLE patients and 1,728 healthy controls (Table 1). The direction of change in all loci was the same as that previously reported [14-22,24]. In the sex-stratified analysis, the 19 loci were associated in women (1,321 SLE patients and 1,188 controls) and 9 in men (136 SLE patients and 540 controls), in part reflecting the decrease in sample size of the male subgroup (Table 2). Comparison of risk allele frequencies between women and men with SLE revealed a significant difference only in rs1143679, the *ITGAM* SNP, which was more frequent in men than in women with SLE. Our results at this locus and at other three loci are in contrast with those reported by Hughes *et al*. [9], who found that the *ITGAM* SNP was not different and the other three loci showed significant differences between women and men with SLE. These differences were an excess of the risk alleles of two of them in men, rs3131379 in *MSH5* (*HLA*) and rs10488631 in *IRF5*, and an excess of the third, rs4963128 in *KIAA1542*, in women. None of these changes were observed in our samples (Table 2).

**Table 2:** **Comparisons of the risk allele frequencies of 19 systemic lupus erythematosus autosomal loci in patients and controls stratified by sex and between men and women with SLE**^**a**^

		**Men**^**b**^				**Women**^**b**^				**SLE men/SLE women**	
**SNP**^**c**^	**Gene locus**	**SLE**	**Control**	**OR (95% CI)**	***P***-**value**	**SLE**	**Control**	**OR (95% CI)**	***P***-**value**	**OR (95% CI)**^**d**^	***P***-**value**
rs2476601	*PTPN22*	0.114	0.070	1.70 (1.09 to 2.64)	0.017	0.097	0.076	1.31 (1.08 to 1.60)	7.2 × 10^−3^	1.19 (0.80 to 1.77)	0.4
rs1801274	*FCGR2A*	0.493	0.474	1.08 (0.82 to 1.41)	0.6	0.511	0.474	1.15 (1.03 to 1.29)	0.011	0.93 (0.72 to 1.20)	0.6
rs2205960	*TNFSF4*	0.257	0.209	1.31 (0.96 to 1.79)	0.09	0.264	0.220	1.27 (1.12 to 1.45)	3.1 × 10^−4^	0.97 (0.72 to 1.29)	0.8
rs7574865	*STAT4*	0.306	0.209	1.67 (1.24 to 2.25)	7.1 × 10^−4^	0.325	0.243	1.49 (1.32 to 1.69)	2.8 × 10^−10^	0.92 (0.70 to 1.20)	0.5
rs6445975	*PXK*	0.311	0.241	1.42 (1.06 to 1.91)	0.018	0.264	0.226	1.23 (1.08 to 1.40)	2.2 × 10^−3^	1.26 (0.96 to 1.66)	0.09
rs17266594	*BANK1*^e^	0.730	0.715	1.07 (0.80 to 1.45)	0.6	0.751	0.708	1.24 (1.09 to 1.41)	7.5 × 10^−4^	0.89 (0.67 to 1.19)	0.4
rs3131379	*MSH5 (HLA)*	0.132	0.071	1.98 (1.30 to 3.02)	1.2 × 10^−3^	0.146	0.072	2.20 (1.82 to 2.66)	9.6 × 10^−17^	0.89 (0.62 to 1.29)	0.5
rs729302	*IRF5*	0.711	0.689	1.11 (0.83 to 1.49)	0.5	0.748	0.691	1.33 (1.17 to 1.50)	1.0 × 10^−5^	0.83 (0.63 to 1.10)	0.2
rs10488631	*IRF5*^e^	0.184	0.111	1.81 (1.26 to 2.61)	1.2 × 10^−3^	0.183	0.097	2.09 (1.76 to 2.48)	1.0 × 10^−17^	1.01 (0.73 to 1.40)	0.96
rs10954213	*IRF5*	0.639	0.638	1.01 (0.76 to 1.33)	0.97	0.685	0.643	1.21 (1.07 to 1.36)	1.9 × 10^−3^	0.82 (0.63 to 1.06)	0.13
rs13277113	*C8orf13-BLK*	0.274	0.250	1.13 (0.84 to 1.53)	0.4	0.311	0.245	1.39 (1.23 to 1.58)	2.8 × 10^−7^	0.84 (0.63 to 1.11)	0.2
rs4963128	*KIAA1542*	0.715	0.645	1.38 (1.03 to 1.85)	0.03	0.696	0.662	1.17 (1.04 to 1.32)	0.010	1.10 (0.83 to 1.45)	0.5
rs1143679	*ITGAM*	0.299	0.169	2.10 (1.54 to 2.85)	1.6 × 10^−6^	0.230	0.153	1.65 (1.42 to 1.90)	1.2 × 10^−11^	1.43 (1.08 to 1.88)	0.011
rs2304256	*TYK2*	0.735	0.723	1.06 (0.79 to 1.44)	0.7	0.770	0.727	1.26 (1.11 to 1.43)	4.8 × 10^−4^	0.83 (0.62 to 1.11)	0.2
rs5754217	*UBE2L3*	0.244	0.209	1.22 (0.89 to 1.67)	0.2	0.254	0.218	1.22 (1.07 to 1.39)	2.8 × 10^−3^	0.95 (0.71 to 1.27)	0.7
rs2230926	*TNFAIP3*	0.070	0.039	1.87 (1.07 to 3.27)	0.026	0.074	0.041	1.87 (1.45 to 2.40)	7.0 × 10^−7^	0.95 (0.58 to 1.55)	0.8
rs573775	*ATG5*	0.256	0.263	0.96 (0.71 to 1.30)	0.8	0.301	0.269	1.17 (1.04 to 1.33)	0.012	0.80 (0.60 to 1.06)	0.12
rs2187668	*HLA-DQA1*	0.184	0.107	1.87 (1.30 to 2.69)	6.0 × 10^−4^	0.201	0.105	2.15 (1.82 to 2.52)	7.4 × 10^−21^	0.89 (0.65 to 1.23)	0.5
rs10798269	*1q25.1*	0.733	0.656	1.20 (0.89 to 1.62)	0.2	0.729	0.680	1.26 (1.12 to 1.43)	1.7 × 10^−4^	1.02 (0.77 to 1.36)	0.9

^a^SLE, Systemic lupus erythematosus; SNP, Single-nucleotide polymorphism. ^b^SLE men = 136; control men = 540; SLE women = 1,321; Control women = 1,188. ^c^The 13 first loci are the same as those studied by Hughes *et al*. [9]. ^d^OR > 1.0 indicates higher frequency of the risk allele in men than in women. ^e^*r*^2^ = 0.94 with rs10516487 used for *BANK1* and *r*^2^ = 0.94 for rs2070197 used for *IRF5*[9].

**Figure 1:**
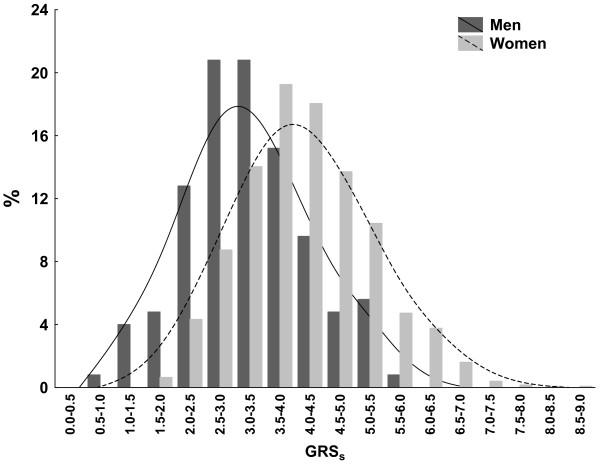
**Sum of genetic risk scores in women and men with systemic lupus erythematosus.** The *y*-axis represents percentages of participants in each of the two groups with the sum of genetic risk scores (GRS_s_) at the indicated intervals along the *x*-axis. This analysis was done with GRS_s_ obtained for each of the 1,247 women and 125 men with systemic lupus erythematosus (SLE) and 100% genotype success at the 19 SLE loci. The GRS_s_ of each patient with SLE is the sum of the products of the natural logarithm of the sex-specific OR by the number of risk alleles at each locus carried by the patient, as described by Hughes *et al*. [9]. Histograms and distance-weighted least-squares fitting lines for women (light gray bars and discontinuous line) and for men (dark gray bars and continuous line) are shown.

GRS_s_ were obtained separately for women and men with SLE using the risk alleles and sex-specific ORs of the 19 loci (Figure 1). They showed very significantly higher values in women than in men (mean GRS_s_ ± SD = 4.15 ± 1.07 vs 3.22 ± 1.0, respectively; *P* < 10^−16^ by Student’s *t*-test). This result was opposite to that observed by Hughes *et al*. [9], that is, higher GRS_s_ in men than in women. The difference was very significant regardless of consideration of the 77.1% nonoverlapping samples (mean GRS_s_ ± SD in women = 4.20 ± 1.07 vs 3.27 ± 0.98 in men; *P* = 8.9 × 10^−16^) or of the 22.9% samples overlapping with those studied by Hughes *et al*. (3.98 ± 1.07 in women vs 3.05 ± 1.02 in men; *P* = 1.3 × 10^−5^). The mean difference in GRS_s_ between the sexes, 0.93, was the same in the two subgroups of patients. This result of the overlapping samples was not reflected in the Hughes *et al.* study, because they were only a minor fraction (7.9%) of the samples included in that study and they were considered together with all others.

Differences in the loci investigated between the two studies include three components: six loci included in our study that were not in the Hughes *et al*. study, thirteen loci shared by the two studies and two loci in the Hughes *et al.* that are absent from our study. The six loci included only in our study showed either no difference or a trend toward higher risks in women (Table 2). Comparison of the GRS_s_ between women and men restricted to these six loci gave a very significant excess of risk load in women (mean GRS_s_ for these six loci = 1.29 ± 0.55 in women and 0.74 ± 0.46 in men; *P* < 10^−16^), which accounted for most of the difference between the two sexes in our study (0.55, or 59% of the 0.93 difference with the 19 loci). The 13 loci that are shared by the two studies also contributed to the contrasting results, but less markedly so (mean GRS_s_ of the 13 shared loci = 2.86 ± 0.82 in women and 2.48 ± 0.77 in men; *P* = 6.6 × 10^−7^). Their effect is reflected in the remaining 41% of the mean difference with the 19 loci (0.38 of the 0.93 difference). Therefore, whereas each of the six loci included only in our study contributed 0.09 to the mean difference in GRS_s_, each of the loci included in both studies contributed only 0.03 to the average score. We cannot exclude the possibility that the two loci included in the previous report that we did not study might have contributed to the very contrasting results.

Two HLA loci accounted for a large fraction of the previously reported increased GRS_s_ in men with SLE [9]. We also included two strongly SLE-associated HLA loci in our analyses (rs3131379 and rs2187668), but they did not account for a particularly large fraction of the difference between sexes, as demonstrated by the similar result obtained after excluding these two SNPs (mean GRS_s_ except HLA = 3.61 ± 0.81 for women and 2.82 ± 0.76 for men with SLE (*P* < 10^−16^), corresponding to 85% of the difference in GRS_s_ with the 19 loci).

## Discussion

Our analysis shows a very significantly higher genetic risk load in women than in men with SLE. This result is in striking contrast to the previous analysis by Hughes *et al*. [9] and highlights important limitations of this type of study that prevent firm conclusions about the relative genetic risk load of the two sexes until these limitations can be addressed.

The most prominent outcome of our study is the lack of reproducibility of the results, both at the level of each locus and at the level of global genetic risk load measured by GRS_s_. At the locus level, lack of reproducibility is well known to have plagued genetic association studies in the past [26], and it has been notably common in studies claiming sex-specific associations [27]. Two of the limitations of these studies are especially relevant here: tolerant significance thresholds that do not account for the multiple tests involved and small sample sizes that lead to imprecision in the estimated effects. Regarding the significance thresholds, none of the loci showed a difference between sexes with *P* < 10^−4^ in the two studies. Therefore, all nonreproducible locus associations will be eliminated if a strict threshold is applied. This limitation does not apply, however, to the comparison of GRS_s_, because only a test was done in each study and the results obtained are very significantly different. In contrast, the second limitation—the decrease in sample size inherent to stratification by sex—has deleterious effects in both the analysis of each locus and the calculation of GRS_s_. The effect at the locus level can be shown by comparing, within each study, the power to detect differences between SLE patients and controls with the power to detect differences between women and men with SLE. Our study has enough power (1 − β > 0.8 for α = 0.05 and risk allele frequency = 0.2) to detect differences with OR = 1.19 in the first comparison, but only for OR > 1.52 in the second. Similarly, the Hughes *et al*. study [9] has enough power for detection with an OR > 1.12 in the patient–control analysis but an OR > 1.31 in the female–male comparison. The decrease in power affects the precision of the OR, which is used to calculate GRS_s_ and, therefore, also has a negative effect in the reproducibility of the GRS_s_ results. Thus, GRS_s_, as is true of other summary parameters, is less variable than the individual OR included in its calculation, but GRS_s_ performance depends on the OR quality, which is determined by sample size and the minor allele frequency of the SNPs [28].

A second notable message derived from the data is that most known SLE loci are not clearly biased to more risk in one of the sexes. In our study, only one of the nineteen SNPs showed a significant difference between women and men with SLE. The risk allele of this SNP (rs1143679) in *ITGAM* was more associated with SLE in men than in women. Similar results were reported by Hughes *et al*. [9], who showed that only four loci (rs1270942 in the HLA, rs3131379 in *MSH5* (*HLA*), rs10488631 in *IRF5*, and rs4963128 in *KIAA1542*) of the fifteen analyzed had significant differences between women and men with SLE. They found that the risk alleles of three of these loci were more common in men than in women with SLE, but the fourth (in *KIAA1542*) was more common in women than in men with SLE. In addition, when the Bonferroni correction is applied for the number of loci analyzed, only the two HLA loci included in the Hughes *et al*. study remain significantly different between the sexes in either of the two studies. Given this lack of clear bias for most SLE loci to excess risk in one of the sexes, differences in estimated global genetic risk load will be sensitive to the inclusion of loci showing small and inconsistent differences, with some showing a small bias toward one sex and others to the opposite sex. The balance between the two components will determine the global outcome. In addition, solving the question of the different genetic risk load in women and men will be difficult until we know a larger fraction of the heritability of SLE than the current 9% to 15% [16,29]. This fraction of heritability has be explained with up to 23 loci, but the most current estimates put the number of loci for complex diseases such as SLE at several hundred [30]. These estimates indicate that many of the yet undiscovered loci will show lower effects than those already known. However, it is clear that there is plenty of room for improvement. Larger coverage of SLE heritability will permit more reproducible assessment of differences in the total genetic burden between the sexes.

The very significant differences (*P* < 10^−16^ and *P* < 5 × 10^−8^) in genetic risk load in the two studies indicate that there are factors other than the imprecision of GRS_s_ leading to their opposite directions. Two factors are likely to have been major contributors: the different sets of loci and the differences between the participants included. Our analysis shows that the different sets of loci in each study were an important factor. The six loci we explored that were not included in the previous study accounted for more than one-half of the excess risk in women, despite representing less than one-third of the analyzed loci. In addition, it is very likely that lack of the HLA SNP rs1270942 in our study also contributed to the contrasting results, because it accounted for the highest risk in men of all the loci included in the Hughes *et al.* study [9]. Regarding the differences between the study participants, they are shown by the significantly higher risk load in women than in men in our study (*P* < 6.6 × 10^−7^), based on the GRS_s_ calculated for the 13 loci shared by the two studies. The differences between the two studies could include genetic heterogeneity affecting both patients with SLE and controls and clinical differences between the patients with SLE. Genetic heterogeneity is especially likely at the HLA SNPs, where large differences, even within subpopulations of the same ethnic group, are common. The two types of heterogeneity, genetic and clinical, have previously been shown to be reflected in the degree of association of SLE loci [11,31-34]. In this regard, we could compare the prevalence of only three SLE classification criteria, but they showed significant heterogeneity between the SLE patients included in the two studies. Men in the Hughes *et al*. study had a higher prevalence of renal disease (OR = 1.7, *P* = 1.2 × 10^−5^) than that in women, but not of serositis or neurologic involvement [9]. In contrast, the men in our study had a higher incidence of serositis than the women did (OR = 1.58, *P* = 0.017), but not renal (OR = 1.23, *P* = 0.3) or neurologic involvement. This type of inconsistency between studies regarding clinical differences between women and men with SLE are common [3-6].

There is an additional point to consider in the interpretation of this type of study: the possibility of ascertainment bias. This artefact is the systematic deviation from the true population value that is attributable to the sampling processes used to find SNPs and estimate their population-specific allele frequencies [35]. As all the SLE loci have been discovered in studies involving either only women or a very dominant fraction of women, they reflect more faithfully genetic susceptibility to SLE in women than in men. Therefore, they are more likely to show stronger associations with women than with men, in cases where the genetic susceptibility of the two sexes is different. The possibility of ascertainment bias means that the increased genetic risk load we found in women cannot be interpreted as a more marked genetic component in SLE susceptibility in women than in men.

## Conclusions

Our results highlight that there is not a uniform trend in the known SLE loci toward higher genetic risk in one or the other of the sexes among Europeans. In addition, our results show the multiple limitations of studies in which investigators aim to establish sex-specific genetic risk loads in SLE. These limitations include the critical role of the assortment of loci that are considered in a particular study; the imprecision in GRS_s_ estimates for men; heterogeneity between sets of participants, including genetic and clinical heterogeneity; and the possibility of ascertainment bias when analyzing loci identified predominantly in women. The limitations could someday be addressed in studies in which researchers include SNPs representing a larger fraction of the SLE genetic component of what is currently known and that have been identified in large samples of both women and men; however, such studies are not currently possible. Therefore, at present, these limitations prevent assessment with confidence of differences in genetic load between women and men with SLE.

## Abbreviations

GRS_s_: Sum genetic risk score; HLA: Human leukocyte antigen; SLE: Systemic lupus erythematosus; SNP: Single-nucleotide polymorphism.

## Competing interest

The authors declare that they have no competing interests.

## Author’s contributions

EAP and AG designed the study and wrote the first draft of the manuscript. EAP and MSG genotyped the samples. EAP, MSG, MC and AG analyzed the data. FJB, AS, MJS, CP, PC, RP, JOR, MM, FNS, MB, NB, GDS, SM, TW, BRL, AK, SR and JJGR provided samples from and clinical and demographic information of the participants. EAP, MSG, MC, FJB, AS, MJS, CP, PC, RP, JOR, MM, FNS, MB, NB, GDS, SM, TW, BRL, AK, SR, JJGR and AG contributed to the interpretation of the data and revised the final version of the manuscript. AG obtained funding, supervised the study and had complete access to all of the data. All authors read and approved the final version of the manuscript.
